# Synthesis and In Vitro Performance of Polypyrrole-Coated Iron–Platinum Nanoparticles for Photothermal Therapy and Photoacoustic Imaging

**DOI:** 10.1186/s11671-017-2337-9

**Published:** 2017-10-18

**Authors:** Thi Tuong Vy Phan, Nhat Quang Bui, Madhappan Santha Moorthy, Kang Dae Lee, Junghwan Oh

**Affiliations:** 1 Marine-Integrated Bionics Research Center, Pukyong National University, Busan, 48513 Republic of Korea; 2 Department of Biomedical Engineering and Center for Marine-Integrated Biotechnology (BK21 Plus), Pukyong National University, Busan, 48513 Republic of Korea; 3 Interdisciplinary Program of Biomedical Mechanical & Electrical Engineering, Pukyong National University, Busan, 48513 Republic of Korea; 4 Department of Otolaryngology – Head and Neck Surgery, Kosin University College of Medicine, Busan, 49267 Republic of Korea

**Keywords:** Iron-platinum nanoparticles, Polypyrrole, Photothermal therapy, photoacoustic imaging, Cancer treatment

## Abstract

Multifunctional nano-platform for the combination of photo-based therapy and photoacoustic imaging (PAI) for cancer treatment has recently attracted much attention to nanotechnology development. In this study, we developed iron–platinum nanoparticles (FePt NPs) with the polypyrrole (PPy) coating as novel agents for combined photothermal therapy (PTT) and PAI. The obtained PPy-coated FePt NPs (FePt@PPy NPs) showed excellent biocompatibility, photothermal stability, and high near-infrared (NIR) absorbance for the combination of PTT and PAI. In vitro investigation experimentally demonstrated the effectiveness of FePt@PPy NPs in killing cancer cells with NIR laser irradiation. Moreover, the phantom test of PAI used in conjunction with FePt@PPy NPs showed a strong photoacoustic signal. Thus, the novel FePt@PPy NPs could be considered as promising multifunctional nanoparticles for further applications of photo-based diagnosis and treatment.

**Electronic supplementary material:**

The online version of this article (10.1186/s11671-017-2337-9) contains supplementary material, which is available to authorized users.

## Background

Over the past decade, many novel therapeutic strategies have been introduced for cancer therapy. In those, photothermal therapy (PTT) gained considerable attention because of its advantages, including high specificity, precise spatial-temporal selectivity, and limited side effects [1, 2]. PTT utilizes the near-infrared region (NIR) photoabsorbers to generate heat for the thermal ablation of cancer cells upon NIR laser irradiation [2]. Taking the advantage of using the laser irradiation with the same wavelength, the NIR photoabsorbers can be used for photoacoustic imaging (PAI)-guided photothermal cancer therapy [3, 4].

Recently, iron–platinum nanoparticles (FePt NPs) have emerged as effective agents for CT/MRI dual modality imaging [5]. FePt NPs display a higher photothermal efficiency than gold nanoparticles in the NIR region [6]. A stronger photoacoustic signal generated by using FePt NPs, in comparison with gold nanoparticles, also was recently demonstrated [7]. Surface modification with polymer is a well-known technique to enhance the biocompatibility and performance of nanoparticles for cancer treatment. Despite their promising properties, there have been a few research efforts on the surface modification of FePt NPs for the biomedical application [8, 9].

The high efficiency of light-to-heat transformation of the nanoscale agents is the most important factor for PTT [10]. Thus, the selected material for the surface modification of FePt NPs should have no negative effect on the light-to-heat transformation of the FePt NP core. Polypyrrole (PPy), which has a strong excitation in the NIR region, has received considerable significance in biomedical applications due to its superior inherent features, including photothermal stability, low cost, and biocompatibility [11, 12]. Recent studies have reported PPy as a high-performance agent for PTT cancer treatment [11, 13] and deep-tissue PAI [12]. In the present work, we developed PPy-coated FePt NPs (FePt@PPy NPs) as novel agents for the combining PTT and PAI. Our expectation when using PPy polymer to coat FePt NPs is to advance the photothermal effect and the biocompatibility of the FePt NPs.

The resulting nanoparticles have shown excellent biocompatibility, photothermal stability, and strong photothermal effect. The MTT assay study revealed that FePt@PPy NPs exhibited an effective cancer therapy. Furthermore, the phantom test of the PAI in conjunction with FePt@PPy NPs showed a strong photoacoustic (PA) signal that is very promising for further applications of the PAI.

## Methods

### Material

Platinum acetylacetonate (Pt(acac)_2_, 97%) was purchased from Acros Organics and used as received. Iron pentacarbonyl (Fe(CO)_5_, 99%), hexadecane-1,2-diol (90%), oleyl amine (80–90%), oleic acid (70%), dioctyl ether (90%), 1-octadecene (90%), 3-mercaptopropionic (3-MPA, 97%), pyrrole (Py, reagent grade, 98%), polyvinyl alcohol (PVA, Mw: 9000–10,000), ammonium persulfate ((NH_4_)_2_S_2_O_8_, 98%), sodium dodecyl sulfate (SDS), potassium ferrocyanide, hydrochloric acid, and 3-(4,5-dimethylthiazol-2-yl)-2,5-diphenyltetrazolium bromide (MTT) were purchased from Sigma-Aldrich and used as received during experiments. Cellular staining reagents including trypan blue, propidium iodide (PI), and Hoechst 33342 were also purchased from Sigma-Aldrich. Dulbecco’s modified Eagle’s medium (DMEM), fetal bovine serum (FBS), penicillin, streptomycin, 1× trypsin, and phosphate-buffered saline (PBS) were purchased from HyClone (South Logan, UT, USA). Distilled water (DI) was used for all experiments.

### Synthesis of FePt@PPy NPs

**Scheme 1:**
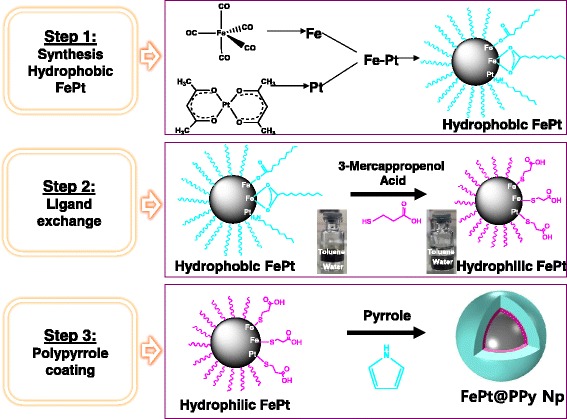
Schematic representation of the synthesis of FePt@PPy NPs

The synthesis of FePt@PPy NPs was performed through three steps which were described in Scheme 1.

#### Step 1—Synthesis of Hydrophobic FePt NPs

The synthesis of hydrophobic FePt NPs was done according to the reported scheme [5]. In short, 97-mg Pt(acac)_2_, 4-mL dioctyl ether, 66-μL Fe(CO)_5_, 195-mg 1,2-hexadecandiol, 100-μL oleyl amine, and 100-μL oleic acid were loaded into a 50-mL three-neck round-bottom flask. The reaction mixture was heated to 240 °C with a heating rate of 15 °C/min under Argon gas. After 30 min, the product was cooled to room temperature. The FePt NPs were collected by centrifugation (15,000 rpm, 30 min) and washed several times with hexane. The final nanoparticle solution was stored in hexane.

#### Step 2—Ligand Exchange

The ligands on the surface of hydrophobic FePt NPs were exchanged with 3-Mercaptopropionic acid (3-MPA) as reported in articles [14]. Moreover, 1 mL of 3-MPA and 1 mL of cyclohexanone were loaded in a centrifuge tube, and then, 0.5 mL of hydrophobic FePt NPs dispersed in hexane (~ 10 mg) was added to the above solution and shaken by using a vortex. After 30 min, the FePt NPs started precipitating, and all nanoparticles precipitated after 1 h. The hydrophilic FePt NPs were collected by centrifugation (3500 rpm, 5 min). The product was washed with cyclohexanone, ethanol, and acetone, respectively. Finally, the hydrophilic FePt NPs diluted in DI with the addition of NaOH.

#### Step 3—Coating Hydrophilic FePt NPs with PPy

Five milligrams of hydrophilic FePt NPs was dissolved in 200-mL barker containing 60-mL DI and was continuously sonicated for 10 min. Then, 6 mL of 40-mM SDS was added to the above solution. Next, 1-g PVA that was completely dissolved in hot water was added to the above solution. The resulting mixture was then stirred at 500 rpm. Next, 10 mL of 6-mM (NH_4_)_2_S_2_O_8_ was added to the stirred solution. After 1 h of equilibration, 6 mL of 100-mM Py was added into the above solution. After several minutes, the solution gradually turned to black. After 2 h of polymerization, the resulting nanoparticles were separated by centrifugation (12,000 rpm, 30 min) and were washed several times with hot water to remove impurities. The obtained FePt@PPy NPs were resuspended with PBS by ultrasonication for 3 min.

### Characterization

The morphology of nanoparticles was observed using field-emission transmission electron microscopy (FETEM; JEM-2100F, JEOL, Japan). The atomic composition was analyze by energy-dispersive spectroscopy (EDS). The chemical functional groups of the nanoparticles were analyzed using a Fourier-transform infrared spectroscopy (FTIR) spectrometer (Perkin Elmer 1320 FTIR spectrophotometer). The nanoparticle diameter was determined by the dynamic light scattering method by using electrophoretic light scattering spectrophotometer (ELS-8000, OTSUKA Electronics Co. Ltd., Japan). UV-Vis-NIR spectra were measured by using UV-Vis-NIR spectroscopy (Thermo Biomate 5 Spectrophotometer). Laser irradiation was performed using a power-tunable 808-nm laser (continuous wave, maximal power = 5 W, Hi-TechOptoelectronics Co., Beijing, China).

### Photothermal Test

For measuring the photothermal performance of as-prepared NPs, a suspension (1 mL) containing the FePt@PPy NPs with specific concentrations (20, 30, 50, 70, 100, and 120 μg/mL) was added into a 12-well plate. Then, each well was exposed by an 808-nm laser at a power density of 1 W/cm^2^ for 5 min. In addition, the increasing temperature of irradiated FePt@PPy NPs at different power densities of the 808-nm laser was also recorded. Briefly, 50-μg/mL FePt@PPy NP solution was irradiated by the NIR laser at the desired power density of 0.5, 1, and 1.5 W/cm^2^ for 6 min. The temperature was recorded by a thermometer (MASTECH, CA, USA) via a thermal fiber.

### Photostability Test

50-μg/mL FePt@PPy NPs was exposed to the 808-nm laser at a power density of 1 W/cm^2^ till the highest temperature was achieved, and then, it was allowed to return to room temperature by turning the laser off. The heating and cooling cycles were repeated six times. The UV-Vis spectrum of the irradiated sample was recorded to compare with the irradiated sample.

### Long-Term Storage Test

The aqueous suspension FePt@PPy NPs at 120 μg/ml concentration was stored at 4 °C for 30 days to evaluate its stability in long-term storage. For the comparison, the UV-Vis absorption spectra and the particles size of FePt@PPy NPs were observed for the 1st day and the last day. In addition, FePt@PPy NPs on different media including DI, DMEM media plus FBS, and PBS were stored at 4 °C for 30 days to evaluate the stability of prepared FePt@PPy NPs.

### Cytotoxicity Assay of FePt-PPy NPs

A standard MTT assay [15] was used to quantify the cell cytotoxicity. The MDA-MB-231 breast cancer cells were used as model cancer cells to test the biocompatibility of FePt@PPy NPs. FePt NP-treated cancer cells were used as a control. The MDA-MB-231 cell line was cultured in a DMEM medium supplemented 10% FBS and 1% antibiotics in a humidified atmosphere at 37 °C and 5% CO_2_. The MDA-MB-231 cells were seeded in 96-well microplate at a density of 1 × 10^4^ cells/well. After 24 h, the DMEM media containing FePt@PPy NPs (or FePt NPs) with different concentrations (0, 20, 30, 50, 70, 100, and 120 μg/mL) were added to cell plates, and the treated cells were then incubated for 48 h. Note that the amount of FePt is the same for the two tested nanoparticles, including FePt NPs and FePt-PPy NPs. Next, 100-μL MTT dissolved in PBS at 0.5 mg/mL was added to each well, and the cell plates were further incubated for 4 h. The dehydrogenase enzyme, which is present in the mitochondria of the alive cells, converted the soluble MTT to insoluble purple formazan. Next, 100 μL DMSO was added to dissolve the insoluble purple formazan. Subsequently, the absorption of purple formazan was recorded at 570 nm using a plate reading spectrophotometer to quantify the percentage of cell viability.

### Cellular Uptake

Prussian blue staining was used to check the cellular uptake of FePt@PPy NPs in the MDA-MB-231 cell [16]. The cells were seeded at a density of 1 × 10^5^ cells/mL in 12-well plates and incubated for 24 h. Next, 200-μg/mL FePt@PPy NPs was added to the cell plates and incubated for another 24 h. After that, the cells were fixed with cold formaldehyde for 15 min. And then, 10% potassium ferrocyanide and 20% aqueous solution of hydrochloric acid (50:50 *v*/*v*) were added to the cell plates and incubated for 1 h. The result was observed using optical microscopy.

### In Vitro Photothermal Therapy

The MTT assay was performed to quantify the efficacy of FePt@PPy NPs on the killing capability of MDA-MB-231 breast cancer cells. Briefly, the MDA-MB-231 cells were cultured in a 96-well microplate at a density of 1 × 10^4^ cells/well. On the next day, the FePt@PPy NP solutions with specific concentration (0, 10, 20, 30, 50, 70, and 100 μg/mL) were added to the cell plates, and the treated cells were incubated for another 24 h. Then, PBS was used to wash the unbound nanoparticles. Subsequently, the microplates were exposed to the NIR laser at a power density of 1 W/cm^2^ for 4 and 6 min, respectively. To obtain the results, the following steps were conducted in accordance with the cell cytotoxicity assay in the “Cytotoxicity Assay of FePt-PPy NPs” section.

Double staining of Hoechst 33342 and PI was also used to detect the damaged and dead cells as a result of the photothermal treatment using FePt@PPy NPs. Concretely, the MDA-MB-231 cells were seeded in a 12-well plate at a density of 1 × 10^5^ cells/well. After 24 h, the cells were treated with the FePt@PPy NPs (0, 50, 70, and 100 μg/mL) and continuously incubated for another 24 h at 37 °C. Next, the unbound nanoparticles were removed by washing gently with PBS. Subsequently, the cell plates were exposed to the NIR laser at a power density of 1 W/cm^2^ for 6 min. Next, the cell culture plates were kept for 24 h in the incubator, and then, the irradiated cells were stained with Hoechst 33342 and PI. Note that 1.5-mL Hoechst 33342 (10 μg/mL) was added in the cell culture plate and then kept in the incubator for 20 min. Then, the cells were washed with three-time PBS to remove the excess stain. Then, the cells were continuously stained with 1.5-mL PI (10 μg/mL) and incubated at room temperature for 5 min. Finally, the cells were again washed with PBS, and the fluorescent images were captured by a fluorescence microscope (Leica Microsystems GmbH, Wetzlar, Germany).

### Animal Experiment

To perform an in vivo test of the photothermal properties of FePt@PPy NPs, a 6-week-aged female BALB/c nude mouse was subcutaneously injected with 100 μL of 100 μg/mL FePt@PPy NPs in PBS. Another nude mouse without injection was used as a control. Afterwards, the injected area of the mice was irradiated with an 808-nm laser at 1 W/cm^2^ for 6 min. The experimental procedures with animals were approved by the animal care and use committee of Pukyong National University and performed according to the guiding principles for the care and use of laboratory animals.

### In Vitro Photoacoustic Imaging

#### PAI Setup

PAI on phantom was performed to evaluate the PA signal of FePt@PPy NPs. Our group has developed the noninvasive PAI system as reported in the previous study [17]. The schematic diagram of PAI setup was shown in Fig. 11. An optical system embedded with a pulsed Nd-YAD Q-switched laser (Surelite III, CA, USA) was employed. The laser was set at 808-nm wavelength and 10-Hz frequency with 5-ns pulse operation. The input optical fiber having a focal length of 50 mm (Thorlabs, Newton, NJ, USA) was connected to a plano-convex lens. The output optical fiber was linked to a focused transducer (Olympus NDT, USA) and adjusted to the illuminated zone’s center. To record PA signals, the data was digitized and stored via a DAQ (data acquisition) system integrated with the laser system. Subsequently, the recorded data was used to reconstruct 2D images of the phantom by a LabVIEW program.

#### Sample Preparation

The PVA phantom was prepared with 8% PVA to mimic the tissue. The preseeded MBA-MD-231 cancer cells were treated with different concentrations of FePt@PPy NPs (50, 100, and 200 μg/mL) for 24 h, and then, the cells were harvested and mixed with 4% gelatin on the phantom (Fig. 12a). Then, the phantom was covered by a small layer of 4% gelatin and allowed to solidify. Finally, the phantom was fixed on the water tank for PAI processing.

## Results and Discussion

### Synthesis and Characterization of FePt@PPy NPs

**Scheme 2:**
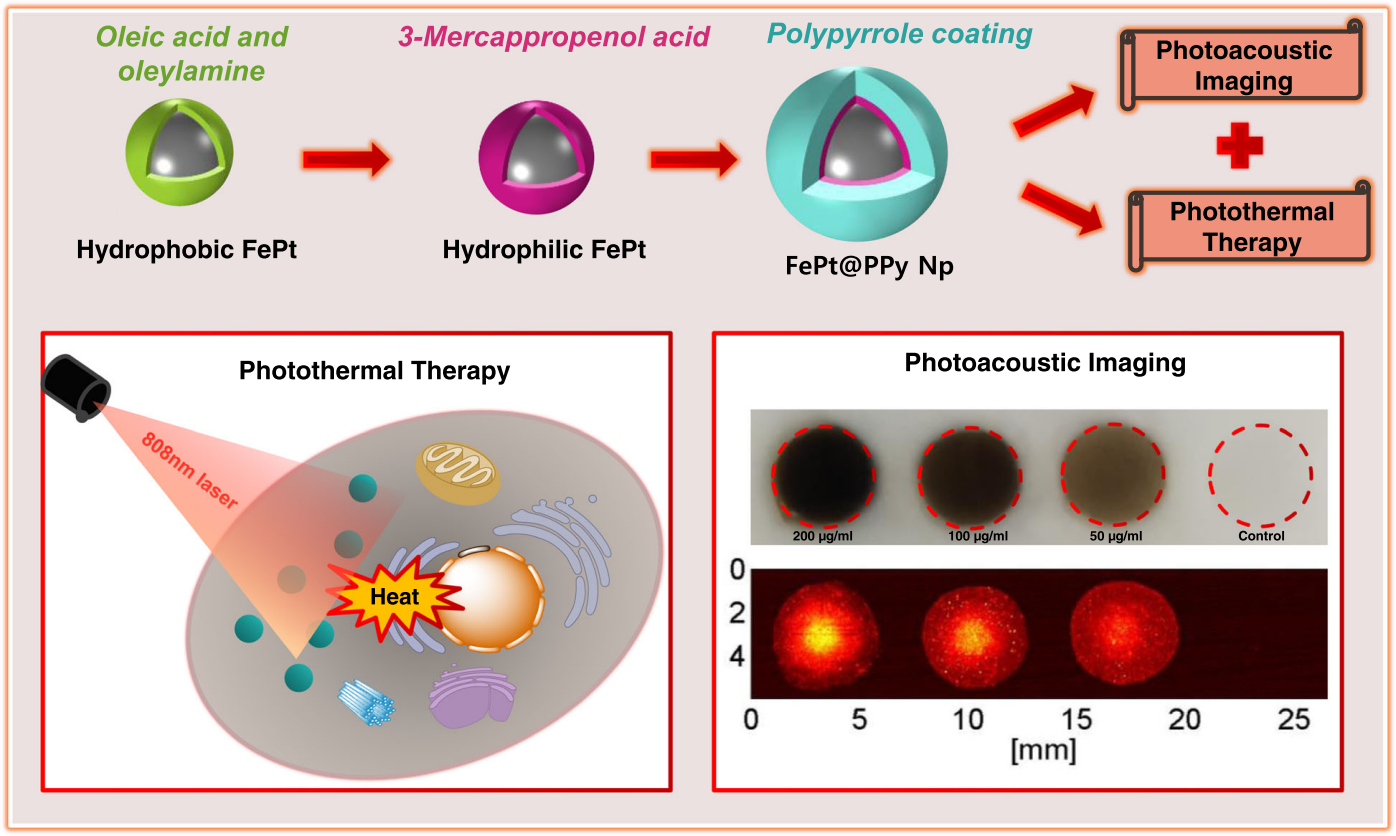
Schematic representation of the synthesis and application of FePt@PPy NPs on photothermal therapy and photoacoustic imaging

The synthesis process of FePt NPs is illustrated in Scheme 1. The EDS analysis of these nanoparticles revealed that the final atomic composition of Fe and Pt are 20 and 80%, respectively (Additional file 1: Figure S1). Hydrophobic FePt NPs were modified with 3-MPA; thus, they become hydrophilic FePt NPs with an average size of 8.3 nm. The hydrophobic FePt NPs disperse in hexane due to the presence of oleic acid and oleyl amine on the surface. However, the particles become soluble in water after ligand exchange. FTIR spectra of hydrophobic FePt NPs and hydrophilic FePt NPs revealed the characteristic bands from absorption ligands of oleic acid, oleyl amine, and 3-MPA on the surface (Fig. 3; Scheme 2) [14, 18]. The FTIR data (Fig. 2) together with the good solubility of hydrophilic FePt NPs in water (Scheme 1, step 2) confirmed the successful ligand exchange process.

**Fig. 1:**
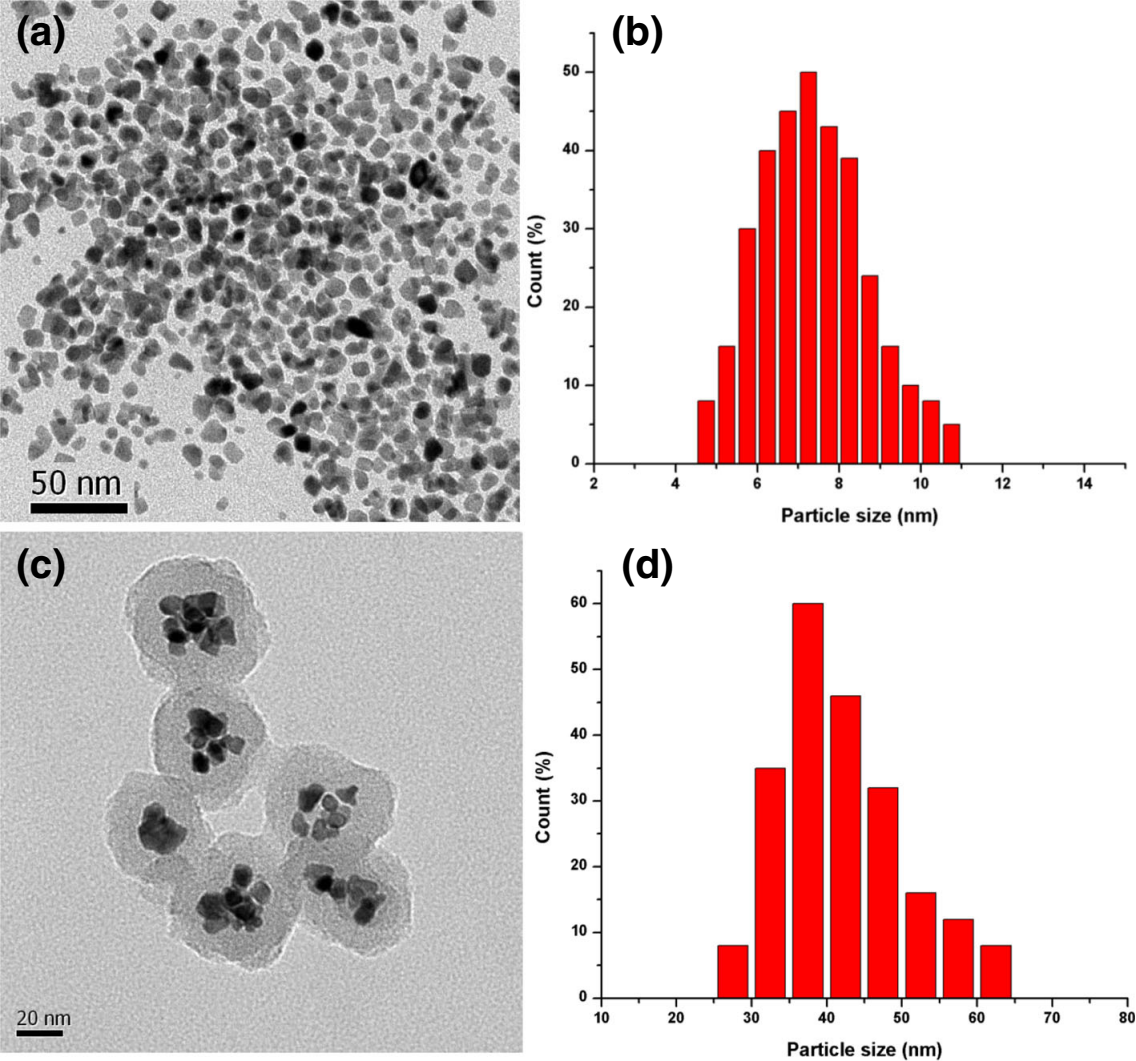
**a** TEM and **b** corresponding size distributions of pure FePt NPs. **c** TEM and **d** corresponding size distributions of FePt@PPy NPs

The FePt NPs were coated with PPy through chemical oxidation polymerization using (NH_4_)S_2_O_8_ as the oxidizing agent and PVA as the stabilizer. The PPy layer was clearly observed by TEM imaging (Fig. 1c) with the thickness about 10 nm, and the average size of FePt@PPy NPs is 42 nm (Fig. 1d). The FTIR of FePt@PPy NPs was also implemented to confirm the coating of PPy NPs by examining the FTIR frequency changes (Fig. 3c). The characteristic peaks of PPy were well analyzed in the previous report [19]. The FTIR vibration bands at 1620 and 1446 cm^−1^ were assigned to C–C and C=C stretching vibrations of a PPy ring. The band at 1236 cm^−1^ was attributed to C–N stretching vibration, and the band at 1076 cm^−1^ indicated the presence of a C–N in-plane deformation mode. Furthermore, the bands at 798 and 600 cm^−1^ were attributed to C–H and N–H in-plane deformation vibration and C–H outer bending vibrations, respectively. The FTIR together with the TEM ensures the successful coating of PPy outer FePt NPs.

**Fig. 2:**
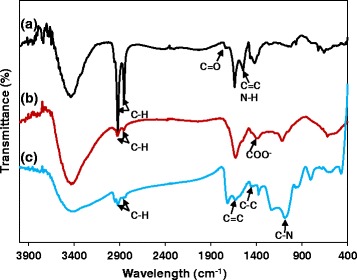
FTIR spectra of (a) hydrophobic FePt NPs, (b) hydrophilic FePt NPs, and (c) FePt@PPy NPs

**Fig. 3:**
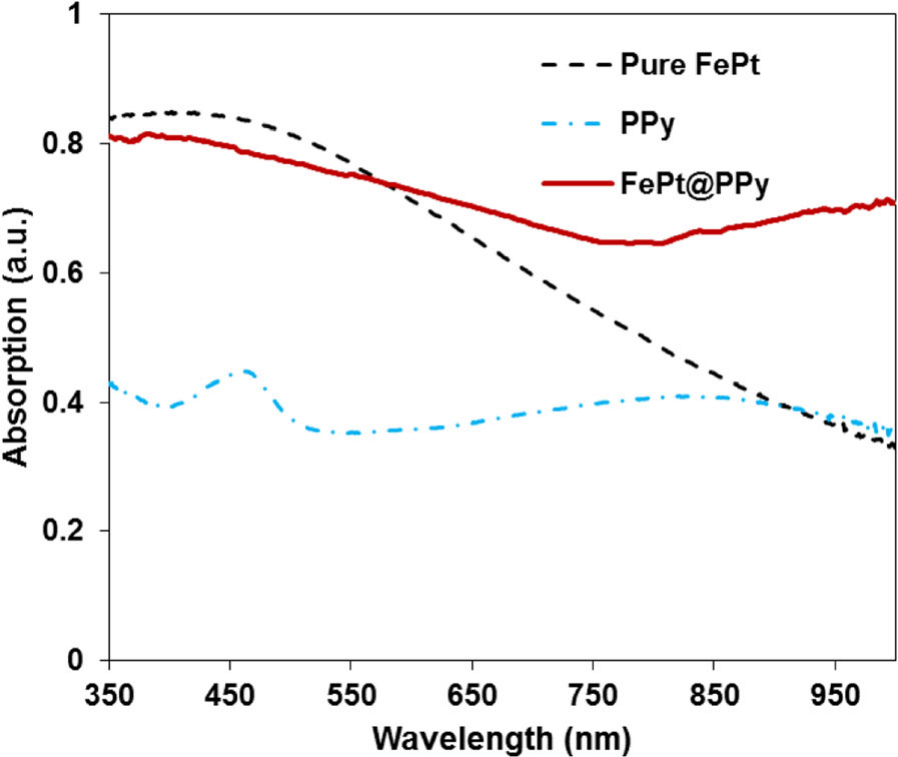
The UV-Vis-NIR spectra of pure FePt, PPy, and FePt@PPy NPs

**Fig. 4:**
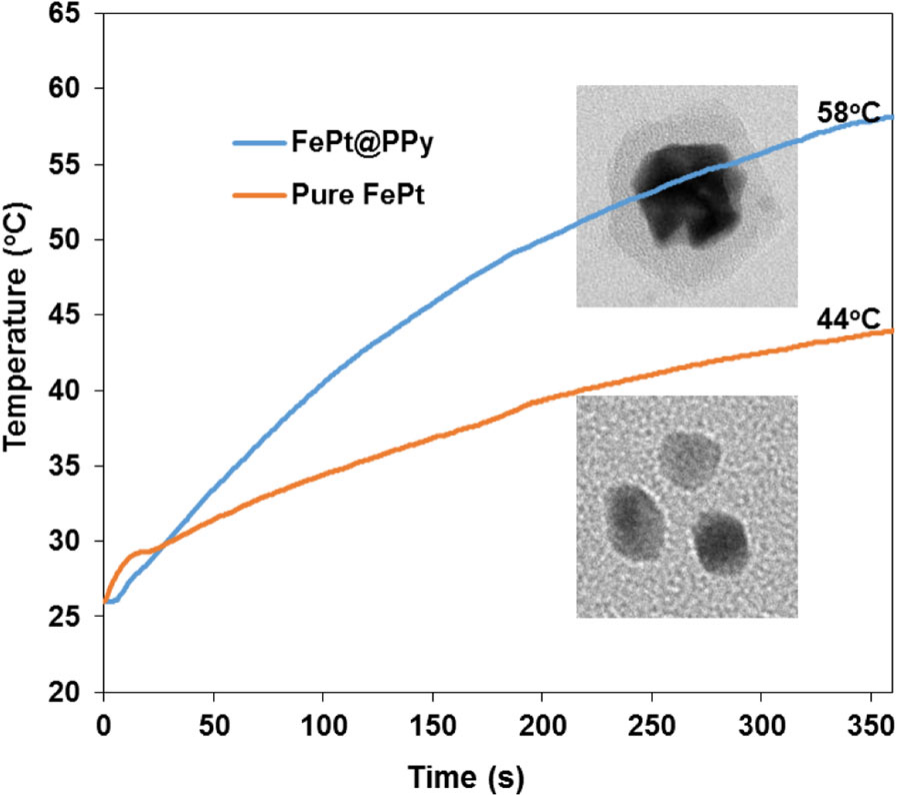
Photothermal heating curve of pure FePt NPs and FePt@PPy NPs with the same amount of FePt. All solutions were irradiated with a 1-W/cm^2^ 808-nm laser for 6 min

**Fig. 5:**
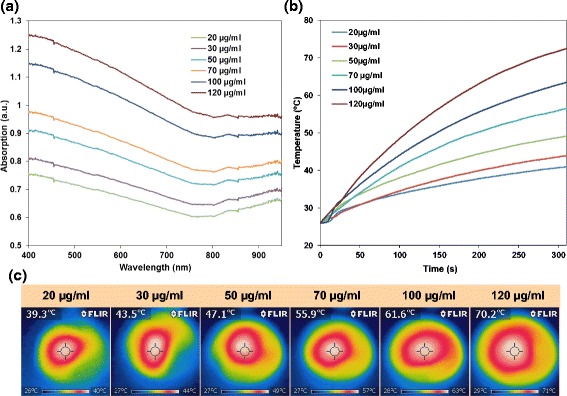
**a** UV-Vis-NIR spectra of different concentrations of FePt@PPy NPs. **b** The photothermal decay of FePt@PPy NPs with different concentrations. **c** The corresponding NIR thermographic images of irradiated samples. All solutions were irradiated with a 1-W/cm^2^ 808-nm laser for 5 min

**Fig. 6:**
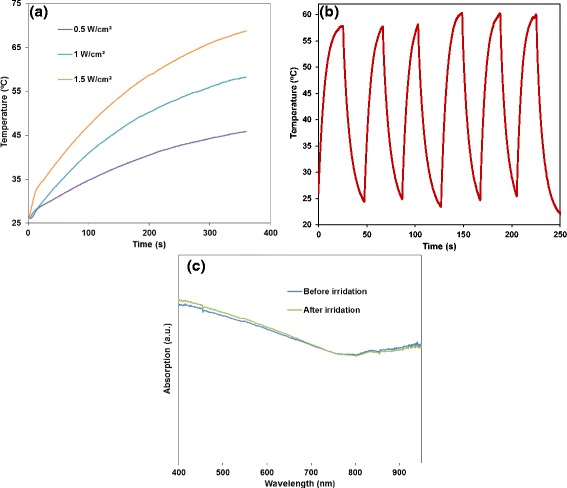
**a** Photothermal behavior of 50 μg/mL of FePt@PPy NPs kept under an 808-nm laser at different power densities for 6 min. **b** The real-time temperature record of six heating/cooling cycles of 50 μg/mL of FePt@PPy NPs under an on/off laser experiment (1 W/cm^2^). **c** UV-Vis-NIR spectra of FePt@PPy NPs before and after irradiation

**Fig. 7:**
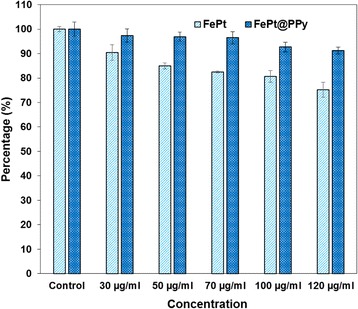
Cell viability (with MTT assay) of MDA-MB-231 cells incubated with FePt and FePt@PPy NPs with different concentrations for 48 h

**Fig. 8:**
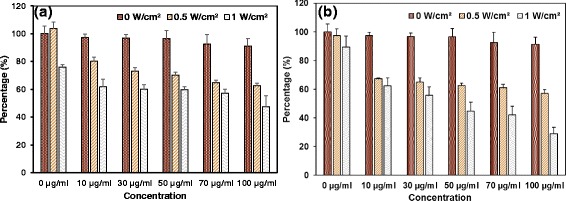
Percentage of cells alive from the cells treated with FePt@PPy NPs under different laser power densities and different irradiation time. **a** Irradiation was performed for 4 min. **b** Irradiation was performed for 6 min

**Fig. 9:**
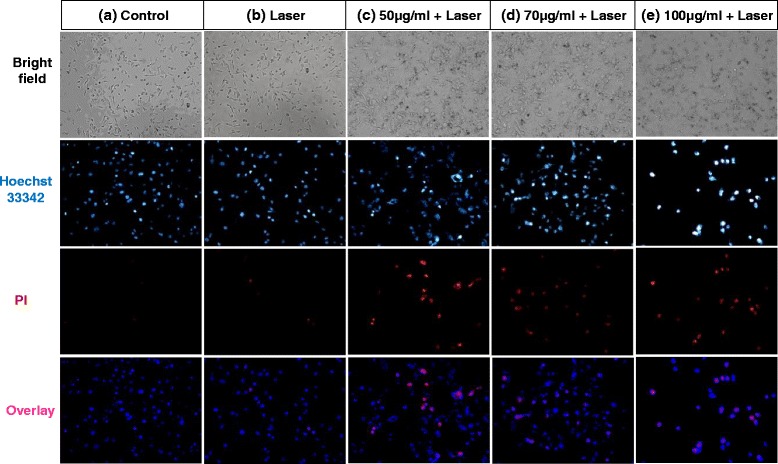
Bright field and fluorescence images of MDA-MB-231 cells under different conditions. **a** Control. **b** Laser only. **c** 50-μg/mL FePt@PPy NPs + laser. **d** 70-μg/mL FePt@PPy NPs + laser. **e** 100-μg/mL FePt@PPy NPs + laser. All solutions were irradiated with a 1 W/cm^2^ 808-nm laser for 6 min

**Fig. 10:**
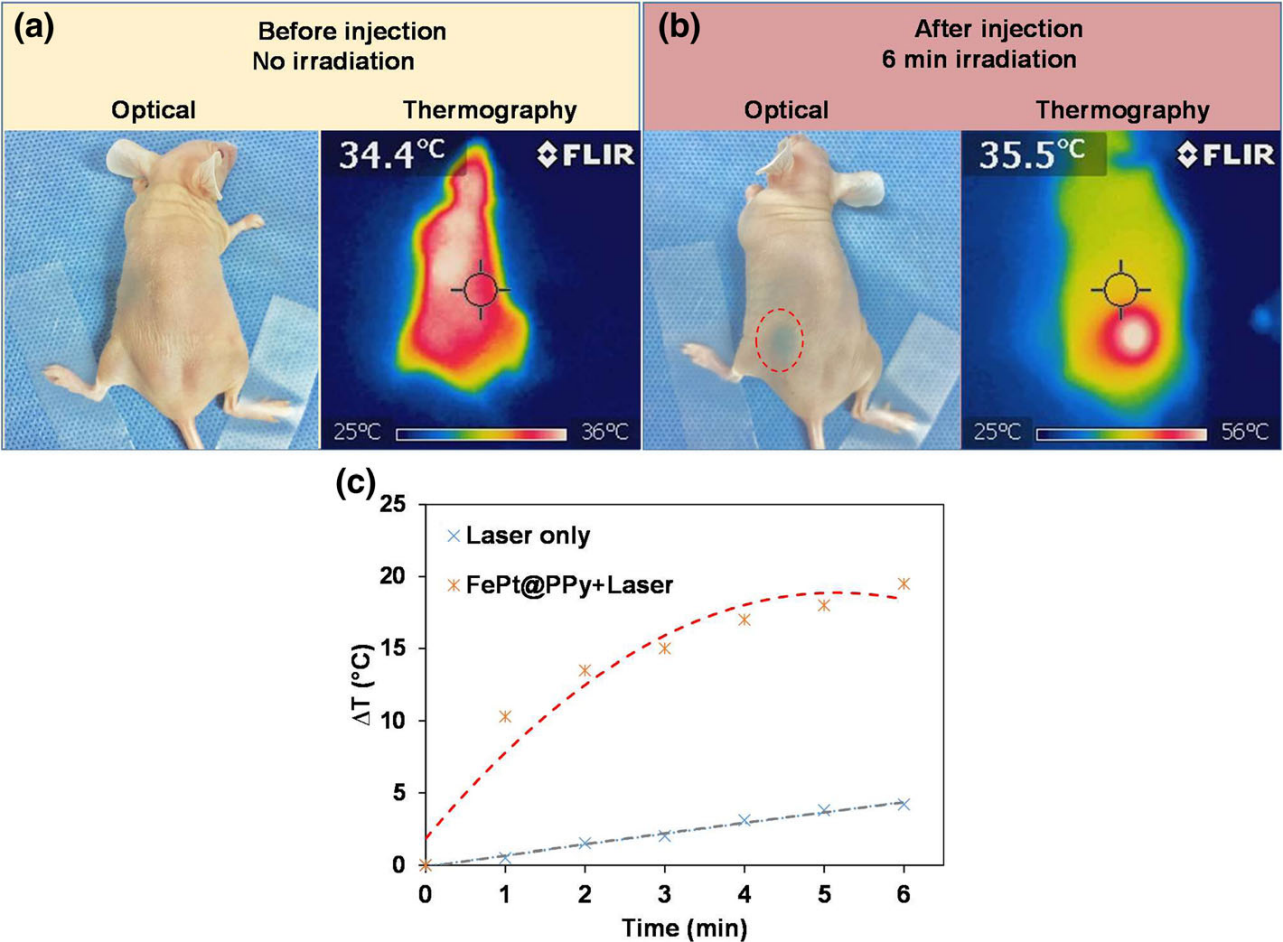
**a** The optical image and the corresponding NIR thermographic images of the nude mouse before injection of FePt@PPy NPs. **b** The left side: the optical image of the nude mouse with subcutaneous injection. The dashed red circle indicates the location of the injection. The right side: the NIR thermographic image of the nude mouse after 6 min under irradiation at an 808-nm laser (1 W/cm^2^). Note that the maximum heating corresponds to the injection site. **c** Temperature change of the skin’s surface at the injection site and in the mice with irradiation at the 808-nm laser (1 W/cm^2^) for 6 min

**Fig. 11:**
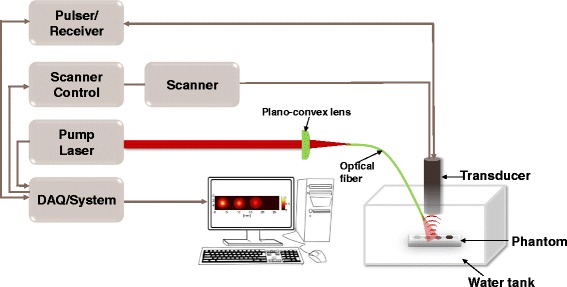
Experimental setup of PAI system

**Fig. 12:**
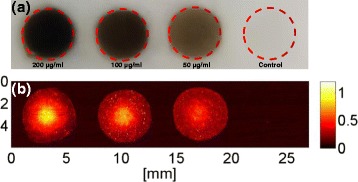
Evaluations on PA responses of FePt@PPy NPs at various concentrations: **a** phantom and **b** corresponding PA images

The UV-Vis-NIR absorption spectra of pure FePt, PPy, and FePt@PPy NPs are given in Fig. 3. The strong absorption at the NIR region was observed for the composite nanoparticles. The absorption spectra of FePt and PPy may together contribute to that of FePt@PPy NPs. The optical properties of FePt@PPy NP aqueous dispersions with different concentrations (from 20 to 120 μg/mL) were also recorded by the UV-Vis-NIR spectroscopy. As plotted in Fig. 4a, with an increase of FePt@PPy NP concentration, the photoabsorption intensity of the entire UV-Vis-NIR region increased.

### Photothermal Performance of FePt@PPy NPs

The photothermal behaviors of pure FePt and FePt@PPy NPs were compared in Fig. 4. Pure FePt and FePt@PPy NPs with the fixed FePt amount were irradiated by an 808-nm laser at a power density of 1 W/cm^2^. FePt@PPy NPs showed excellent photothermal behavior in comparison with pure FePt NPs. This data indicated that the PPy layer enhanced the photothermal efficacy of the whole system.

As shown in Figs. 5a, under the same NIR laser condition (5 min, 1 W/cm^2^), the temperature of the solution containing 20 μg/ml FePt@PPy NPs increased from 25 to 39.3 °C while that containing 120 μg/ml FePt@PPy NPs quickly reached 71 °C. In addition, thermographic images (Fig. 5c) indicated the photothermal effective conversion of the sample containing irradiated FePt@PPy NPs with an 808-nm laser. FePt@PPy NPs (50 μg/mL) were exposed to NIR laser irradiation at different laser power densities of 0.5, 1.0, and 1.5 W/cm^2^ for 6 min, and the resulting temperatures were 41.1, 51.3, and 59.4 °C, respectively. These experimental results revealed that the exposure time, the concentration of nanoparticles, and the laser power intensity are important parameters that significantly influence the photothermal performance of FePt@PPy NPs.

### Photothermal Stability Tests of FePt@PPy NPs

Besides the strong photothermal transduction, the photostability of nanoparticles is important in PTT. FePt@PPy NP solution of 50 μg/mL was irradiated with the 808-nm NIR laser at 1.0 W/cm^2^ until the solution reached the highest temperature, then cooling down naturally to room temperature by turning off the laser. After six cycles of heating and cooling, the thermal curve of FePt@PPy NPs remained almost the same for each cycle (Fig. 4d). The UV-Vis-NIR spectra before and after laser exposure are shown in Fig. 6c. No significant change was observed for the entire spectra. The above results indicated good photothermal stability of FePt@PPy NPs for a long period of NIR laser irradiation.

### Long-Term Storage Test

The particle size and the UV-Vis-NIR absorption spectra of the prepared nanoparticles were monitored during the 30 days of storage. Firstly, no aggregation was observed in all solution containing FePt@PPy NPs (Additional file 1: Figure S3a). Secondly, FePt@PPy NPs in cell culture media at 120 μg/mL concentration did not show any significant change in its UV-Vis-NIR spectra (Additional file 1: Figure S3b) after the 30 days of storage. In addition, the average particle size of FePt@PPy NPs nearly remained unchanged during the long-term storage (Additional file 1: Figure S3c). All the above results proved evidently the stability of the prepared nanoparticles.

### In Vitro Cell Cytotoxicity Assay

For the cancer treatment, nanoparticles should have the excellent biocompatibility. As shown in Fig. 7, the MDA-MB-231 breast cancer cells were treated with pure FePt and FePt@PPy NPs with different concentration and incubated for 48 h. No significant cytotoxicity of the FePt@PPy NPs was observed even at the highest tested concentration (120 μg/mL), and the cell viability of MDA-MB-231 breast cancer cells was still higher than 95%. To the pure FePt NPs, 120 μg/mL irradiated nanoparticles killed 20% cancer cell. This result indicated that the coating of PPy layer improved the biocompatibility of FePt NPs, and FePt@PPy NPs can be considered as a nontoxicity material.

### Cellular Uptake

Prussian blue staining, which bases on the reaction of iron and potassium ferrocyanide in acid solution, was performed to detect the cellular uptake of FePt@PPy NPs. As shown in Additional file 1: Figure S2, most of the cells were stained with blue stains inside the cells, indicating cellular uptake of FePt@PPy NPs.

### In Vitro Photothermal Therapy

The standard MTT assay was performed to evaluate the efficacy of irradiated FePt@PPy NPs on the killing capability of MDA-MB-231 breast cancer cells. First, the cancer cells were incubated with different concentration of FePt@PPy NPs for 24 h and then exposed to the 808-nm laser at 1 W/cm^2^ for 4 min. As shown in Fig. 8, the percentage of cell viability was gradually decreased when the concentration of the treated nanoparticles was increased. Approximately 50% of cells died at a 100 μg/mL concentration of irradiated FePt@PPy NPs. In order to kill more cancer cells, the irradiation time was increased up to 6 min. With 100 μg/mL concentration, approximately 70% of dead cells were observed. A comparison of the photothermal therapy performance between the proposed system and some reported nanoparticles was conducted in Additional file 1: Table S1. It is found that the proposed system shows comparable capability in killing cancer cells (i.e., 70% cell death) with quite low nanoparticle concentration (i.e., 100 μg/mL) under relatively weak power density condition (i.e., 1 W/cm^2^) and short irradiation time (i.e., 6 min).

In addition, by using the fluorescence imaging technique of five groups, we conducted experiments on the cancer cells to consider the killing capability of the prepared nanoparticles: the control groups (only cells), the laser-only group (cells were exposed to the 808-nm laser), the 50-μg/mL FePt@PPy NPs + 808-nm laser (cells were treated with 50-μg/mL of FePt@PPy NPs and exposed to the 808-nm laser), the 70-μg/mL FePt@PPy NPs + 808-nm laser (cells were treated with 50-μg/mL of FePt@PPy NPs and exposed to the 808-nm laser), and the 100-μg/mL FePt@PPy NPs + 808-nm laser (cells were treated with 50-μg/mL of FePt@PPy NPs and exposed to the 808-nm laser).

Double staining of Hoechst 33342 and PI was used to explore the damaged and dead cells. Hoechst 33342 is a DNA dye, which can be permeable in both dead and viable cells [20]. The changes in the size and shape of nuclei of the Hoechst 33342 stained cells can be observed under fluorescence microscopy. With the apoptosis cells, Hoechst 33342 will make the condensed chromatin brighter than that in a normal cell. PI dye also binds to DNA, but it only permeates through the membrane of damaged and dead cells [21]. Thus, double staining can differentiate between dead cells and live cells by each treatment method.

As shown in Fig. 9, the cancer cells exposed to the NIR laser in the presence of the FePt@PPy NPs emit strong fluorescence, whereas the slight fluorescence is emitted by cancer cells in the absence of the nanoparticles. Only a few dead cells with the red nuclei were observed in the control and laser-only group (Fig. 9a, b). In contrast, many cells in the FePt@PPy NPs + 808-nm laser groups died and displayed red nuclei, as observed in Fig. 9c–e. After incubation for 24 h, some dead cells lost the binding ability and were washed out of the cell disk. Therefore, the intensity of cancer cells in the 100-μg/mL of FePt@PPy NPs + 808-nm laser group was less than the others. Conclusively, almost cancer cells which were treated with 100-μg/mL of FePt@PPy NPs was destructed after being exposed to the 808-nm NIR laser at a power density of 1.0 W/cm^2^.

### In Vivo Laser Heating Experiment

The potential ability of FePt@PPy NPs for laser-induced heating was finally tested in an animal model. The nude mouse was subcutaneously injected with 100 μL of an aqueous FePt@PPy (100 μg/mL) NPs in PBS. Figure 10a presents the optical and NIR thermographic images of the nude mouse before injection, pointing out the temperature of mouse surface’s skin is about 36 °C. Fig. 10b (left side) shows an optical image of the mouse in which the injection site is indicated by a dashed red circle. The injected area was irradiated with the 808-nm laser at 1 W/cm^2^ for 6 min, and the NIR thermographic image of this mouse is shown in Fig. 10b (right side). The temperature of the skin’s surface was continuously monitored with an NIR thermographic camera. The time evolution of the surface temperature during the 6 min irradiation is shown in Fig. 10c, figuring out a temperature increment of the skin about 19 °C. From that, we can see clearly that the injected FePt@PPy NP area with laser irradiation produced a high temperature, as required for tumor ablation. Moreover, the heating area was found to be well localized at the injection site as shown in the NIR thermographic image (Fig. 10b, right side). Conclusively, with the excellent laser-induced heating properties, FePt@PPy could be a novel promising agent for photothermal therapy.

### In Vitro Photoacoustic Imaging

The top-view image of the phantom filled with pretreated cancer cells is shown in Fig. 12a. The corresponding PAI acquired at the 808-nm laser from the sample in Fig. 12a is illustrated in Fig. 12b.

PAI is an emerging imaging modality and can be used to assist phototherapy [22]. All the samples containing pretreated cells were clearly visible, whereas the controlled samples with 4% gelatin did not produce any PA signal. The magnitude of the PA signal was increased when the concentration of nanoparticles increased. The ability to image FePt@PPy NPs inside phantom with the PAI system is very promising for image-guided photo-induced cancer therapy. The laser system for PAI, which was used in conjunction with FePt@PPy NPs, also showed the potential for future implementations.

## Conclusions

In this study, we developed the photoabsorber FePt@PPy NPs and evaluated their efficiency on in vitro PTT and PAI (Scheme 2). The prepared FePt@PPy NPs showed many good properties for PTT and PAI including excellent biocompatibility, photothermal stability, and high NIR absorbance. Moreover, in vitro investigation confirmed the effectiveness of the FePt@PPy NPs in killing the cancer cells under the NIR laser. So far, the phantom test of PAI used in conjunction with FePt@PPy NPs showed a strong PA signal. Owing to their good properties, the novel FePt@PPy NPs could be considered as promising multifunctional nanoparticles for further applications in PTT and PAI.

## Additional file

Additional file 1: Synthesis and In vitro Performance of Polypyrrole coated Iron–Platinum Nanoparticles for Photothermal Therapy and Photoacoustic Imaging. (DOCX 4483 kb)
