# Supplementary material for: Synthesis and In Vitro Performance of Polypyrrole-Coated Iron–Platinum Nanoparticles for Photothermal Therapy and Photoacoustic Imaging
Source: Nanoscale Res Lett. 2017 Oct 18;12:570. doi: 10.1186/s11671-017-2337-9 (PMC5647319; doi:10.1186/s11671-017-2337-9)
Supplement: Additional file 1: — Synthesis and In vitro Performance of Polypyrrole coated Iron–Platinum Nanoparticles for Photothermal Therapy and Photoacoustic Imaging. (DOCX 4483 kb) [file 11671_2017_2337_MOESM1_ESM.docx]

Additional file

Synthesis and *In vitro* Performance of Polypyrrole coated Iron–Platinum Nanoparticles for Photothermal Therapy and Photoacoustic Imaging

Thi Tuong Vy Phan, Bui Nhat Quang, Madhappan Santha Moorthy, Kang Dae Lee, and Junghwan Oh

Table S1: The list of some reported nanoparticles with their photothermal performances

| Nanoparticle | Nanoparticle exposure | Cell line | NIR conditions | Cell death | Ref. |
| --- | --- | --- | --- | --- | --- |
| FePt@PPy NPs | 100 μg/mL, 24 h | MDA-MB-231 cells | 1 W/cm^2^, 808 nm, 6 min | 70% cell death | This study |
| Glucose-functionalized gold nanoprisms, | 100 μg/mL, overnight | MEF | 5 W/cm^2^, 1064 nm, 10 min | 58% cell death | ([1](#_ENREF_1)) |
| Polypyrrole-coated gold nanoparticles | 125 μg/mL, 4h | Hela cells | 1.63 W/cm^2^, 808 nm, 3 min | 80 % cell death | ([2](#_ENREF_2)) |
| Polypyrrole-coated flower-like palladium nanoparticles | 50 μg/mL, 24h | Hela cells | 1 W/cm^2^, 808 nm, 10 min | 25% cell death | ([3](#_ENREF_3)) |
| Polypyrrole nanoparticles | 0~100 μg/mL, 24h  250 μg/mL, 24h | Hela cells | 6 W/cm^2^, 808 nm, 10 min | Nearly 20% cell death  80 % cell death | ([4](#_ENREF_4)) |
| Titanium oxide nanoparticles | 125 μg/mL, 4h | 4T1 cells | 1 W/cm^2^, 808 nm, 5 min | 75% cell death | ([5](#_ENREF_5)) |
| Hyaluronan-modified  iron oxide nanoparticles | 200 μg/ml, 24h | MDA-MB-231 cells | 2 W/cm^2^ ,808 nm, 10 min | 75% cell death | ([6](#_ENREF_6)) |
| Fluorescent Iron oxide  composite nanoparticles | 600 μg/ml, 24h | Hela cells | 2 W/cm^2^, 808 nm, 10 min | 80% cell death | ([7](#_ENREF_7)) |
| MoS_2_/Fe_3_O_4_ Nanotheranostic | 200 μg/ml, 24h | Hela cells | 1 W/cm^2^ ,808 nm, 10 min | 94.7% cell death | ([8](#_ENREF_8)) |
| Porphyrin functionalized graphene oxide | 100 μg/ml, 24h | U87-MG brain tumor cells | 2.5 W/cm^2^, 808 nm, 10 min | 76% cell death | ([9](#_ENREF_9)) |


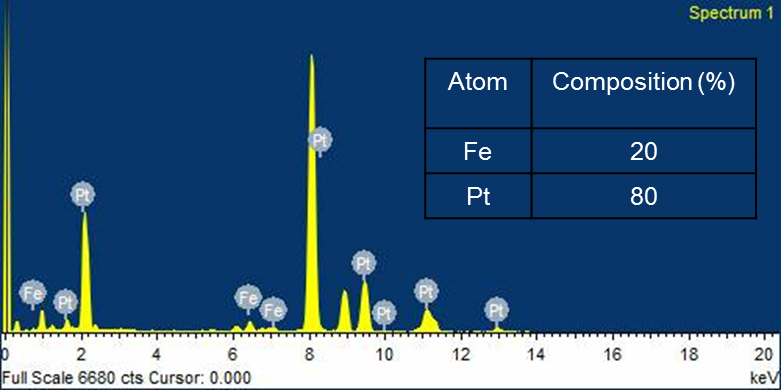


Figure S1: EDS spectra of FePt@PPy NPs.


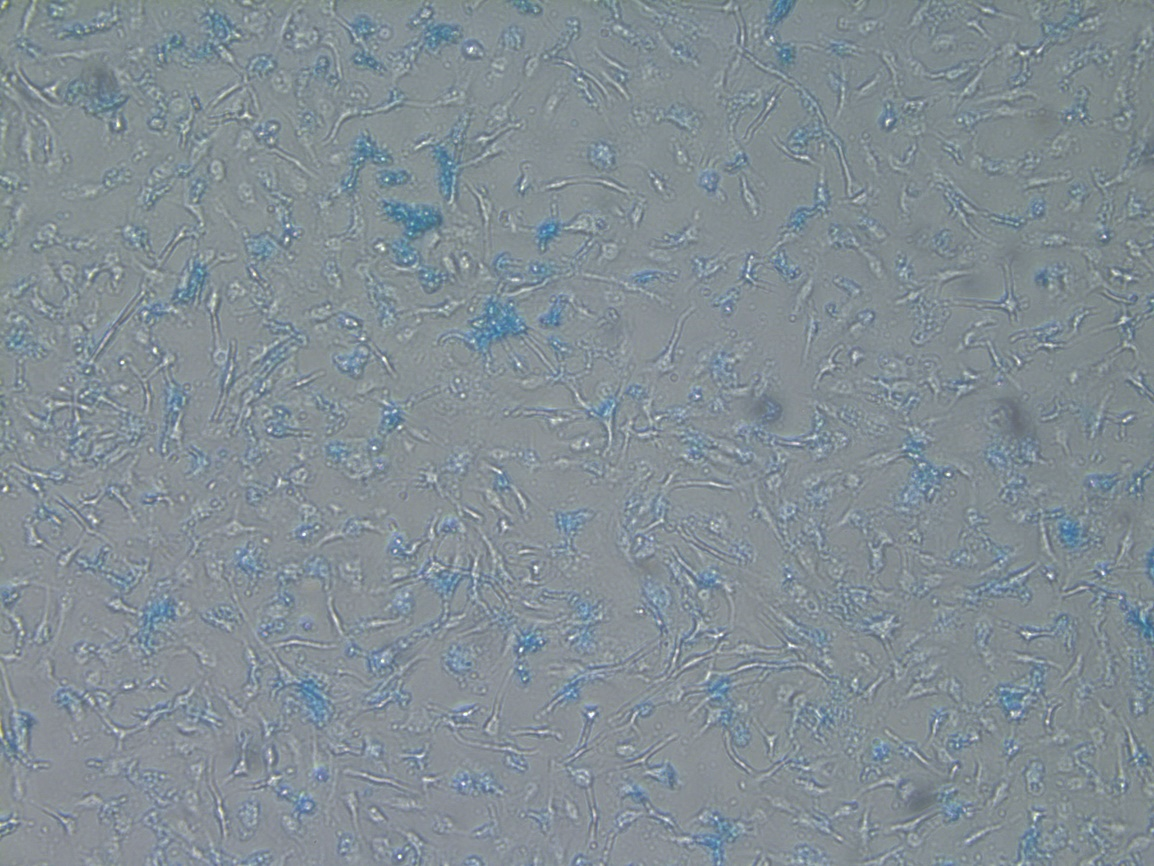


100 µm

Figure S2: Cellular uptake of FePt@PPy NPs by Prussian blue staning. The MDA-MB-231 cells were incubated with 200 μg/mL FePt@PPy NPs for 24 h.

(a)

(c)

(b)

Figure S3: (a) The photos of FePt@PPy NPs in different media after 30 days storage, (b) The UV-Vis-NIR absorption spectra of FePt@PPy NPs at the first day and the 30^th^ day of long-term storage test, (c) The particles size of FePt@PPy NPs at the first day and the 30^th^ day of long-term storage test.

References:

1. Pérez-Hernández M, del Pino P, Mitchell SG, Moros M, Stepien G, Pelaz B, et al. Dissecting the Molecular Mechanism of Apoptosis during Photothermal Therapy Using Gold Nanoprisms. ACS nano. 2015;9(1):52-61.

2. Li J, Han J, Xu T, Guo C, Bu X, Zhang H, et al. Coating Urchinlike Gold Nanoparticles with Polypyrrole Thin Shells To Produce Photothermal Agents with High Stability and Photothermal Transduction Efficiency. Langmuir. 2013;29(23):7102-10.

3. Liu Y, Wang D-D, Zhao L, Lin M, Sun H-Z, Sun H-C, et al. Polypyrrole-coated flower-like Pd nanoparticles (Pd NPs@PPy) with enhanced stability and heat conversion efficiency for cancer photothermal therapy. RSC Advances. 2016;6(19):15854-60.

4. Zha Z, Yue X, Ren Q, Dai Z. Uniform polypyrrole nanoparticles with high photothermal conversion efficiency for photothermal ablation of cancer cells. Advanced materials (Deerfield Beach, Fla). 2013;25(5):777-82.

5. Ou G, Li Z, Li D, Cheng L, Liu Z, Wu H. Photothermal therapy by using titanium oxide nanoparticles. Nano Research. 2016;9(5):1236-43.

6. Yang R-M, Fu C-P, Fang J-Z, Xu X-D, Wei X-H, Tang W-J, et al. Hyaluronan-modified superparamagnetic iron oxide nanoparticles for bimodal breast cancer imaging and photothermal therapy. International Journal of Nanomedicine. 2017;12:197-206.

7. Han X, Deng Z, Yang Z, Wang Y, Zhu H, Chen B, et al. Biomarkerless targeting and photothermal cancer cell killing by surface-electrically-charged superparamagnetic Fe3O4 composite nanoparticles. Nanoscale. 2017;9(4):1457-65.

8. Yu J, Yin W, Zheng X, Tian G, Zhang X, Bao T, et al. Smart MoS2/Fe3O4 Nanotheranostic for Magnetically Targeted Photothermal Therapy Guided by Magnetic Resonance/Photoacoustic Imaging. Theranostics. 2015;5(9):931-45.

9. Su S, Wang J, Wei J, Martinez-Zaguilan R, Qiu J, Wang S. Efficient photothermal therapy of brain cancer through porphyrin functionalized graphene oxide. New Journal of Chemistry. 2015;39(7):5743-9.
